# Comparison of the acute ocular manifestations of Stevens-Johnson syndrome and toxic epidermal necrolysis in Chinese eyes: a 15-year retrospective study

**DOI:** 10.1186/s12886-017-0464-9

**Published:** 2017-05-12

**Authors:** Loraine L. W. Chow, Kendrick C. Shih, Johnny C. Y. Chan, Jimmy S. M. Lai, Alex L. K. Ng

**Affiliations:** 1 Department of Ophthalmology, Queen Mary Hospital, Hospital Authority, Pok Fu Lam, Hong Kong; 2 Department of Ophthalmology, The University of Hong Kong, Room 301, Level 3, Block B, Cyberport 4, 100 Cyberport Road, Pok Fu Lam, Hong Kong; 3 Division of Dermatology, Department of Medicine, The University of Hong Kong, Pok Fu Lam, Hong Kong

**Keywords:** Ocular surface disease, Severe cutaneous adverse reactions, Stevens-Johnson syndrome, Toxic epidermal necrolysis, Asians

## Abstract

**Background:**

Stevens-Johnson syndrome (SJS) and toxic epidermal necrolysis (TEN) are rare but life-threatening conditions that initially affect the skin and mucous membranes. The aim of this study was to compare the acute ocular manifestations between SJS and TEN.

**Methods:**

The initial presenting ophthalmic records of patients with either SJS (<30% body surface area involvement) or TEN (> = 30% involvement), who were treated at one tertiary burn center in Hong Kong between 1999 and 2014, were retrospectively analyzed and compared.

**Results:**

A total of 20 SJS and 12 TEN cases were included. All were drug-induced. The patient demographics and treatment received were comparable. Overall, 40% of SJS and 75% of TEN patients had acute ocular surface inflammation. When comparing the two groups, there was a significant difference in the number of cases with mild involvement (5% in SJS, 42% in TEN, *p* = 0.01), while no statistically significant differences were found (*p* > 0.05) comparing between the moderate (15% in SJS, 0% in TEN) and severe groups (20% in SJS, 33% in TEN).

**Conclusions:**

Ocular surface inflammation was common during the acute phase in both SJS and TEN. TEN had a significantly higher number of cases with mild ocular involvement when compared with SJS, but no significant difference between the number of moderate and severe cases between the two groups.

## Background

Stevens-Johnson syndrome (SJS) and toxic epidermal necrolysis (TEN) are acute exfoliating diseases of the skin and mucosa and represent different ends of the spectrum of the same clinical entity of severe cutaneous adverse reactions. Since 1993 they have been differentiated by total body surface area (BSA) of involvement, with SJS representing the mild end of the spectrum (<10% BSA involvement), TEN forming the most extensive form of the disease (> = 30% involvement) and SJS/TEN overlap in cases with 10 to 30% involvement [1–3]. SJS and TEN are characterized histologically by widespread keratinocyte death and epidermal necrosis resulting in splitting of sub-epidermal layers, resulting clinically in tissue loss of the skin and mucous membranes [4]. Although rare, the conditions result in significant morbidity and mortality [5].

Ophthalmic involvement is common in patients with SJS and TEN, and the acute ocular involvement is reported to occur in 50 to 88% of cases [6, 7]. For affected patients, acute ocular surface inflammation results in devastating long-term sequelae, including dry eye disease, recurrent or persistent corneal epithelial defects, conjunctival scarring, symblephera formation, cornea limbal stem cell deficiency and corneal scarring [6, 8–12]. Currently, the evidence on whether the extent of ocular involvement is the same between SJS and TEN, or worse in either one of the entities, is still conflicting [6, 13–15]. One recent study reported that the SCORTEN value, which is a severity-of-illness score for SJS and TEN that predicts overall mortality, did not correlate with the severity of eye involvement in the acute setting [7]. The aim of this study is to compare the acute ocular surface manifestations of SJS and TEN among in-patients at a tertiary burn care center in Hong Kong over a 15-years period.

## Methods

This is a retrospective cross-sectional study using initial presenting case records of all consecutive patients, who were admitted as inpatients with a diagnosis of either SJS or TEN between the 1st of January 1999 and the 31st of December 2014 at the Queen Mary Hospital, Hong Kong. The study was conducted in accordance with the tenets of the Declaration of Helsinki and was approved by the Institutional Review Board of the University of Hong Kong/Hospital Authority Hong Kong West Cluster. Diagnosis was made by dermatologists based on clinical history or skin biopsy results and patients were classified by the criteria outlined by Bastuji-Garin et al. [1] In our center, patients with less than 30% BSA involvement were all grouped as SJS or SJS/TEN overlap and those with 30% or more BSA involvement as TEN. Patients without available ophthalmic assessment records were excluded from analysis.

Patient data, including demographics, ocular slit lamp examination findings and specific treatment received, were extracted from patient records. Most studies considered the acute stage of the disease as the time of onset of the skin changes [3, 6]. In this study, the acute phase referred to the time period when the patient was admitted to the burn intensive care unit, which closely followed the onset of skin changes. The primary outcome measure was severity of acute phase ophthalmic involvement, which was classified into mild, moderate and severe using clinical parameters on ocular slit lamp examination described by Power et al. [7, 15, 16] Mild ocular involvement comprises any or all of the following: eyelid edema, eyelid skin involvement including denudation and desquamation, mild corneal involvement (punctate fluorescein staining), mild conjunctival injection, mucous discharge, or chemosis. Moderate involvement comprises membranous conjunctivitis, epithelial defects with more than 30% healing with medical treatment, corneal ulceration, or corneal infiltrates. Severe involvement comprises acquired eyelid malpositions, symblepharon formation, non-healing corneal epithelial defects, visual loss or conjunctival fornix foreshortening. We have also classified the severity using another more recent grading system proposed by Sotozono et al. [17] This acute ocular severity scoring system mainly focused on the ocular surface inflammation and epithelial necrosis or apoptosis. Using this system, Grade 1 included eyes with conjunctival hyperemia, which indicated ocular surface inflammation, Grade 2 included eyes with pseudo-membrane formation or presence of epithelial defects, and Grade 3 included eyes with both pseudo-membrane formation and epithelial defects. Secondary outcome measures include the culprit agent and any specific systemic or local treatment received (other than the supportive treatment), including the use of intravenous immunoglobulin or amniotic membrane transplantation.

Statistical analyses were performed using IBM/SPSS software version 21 (IBM/SPSS Inc., Chicago, IL, USA). Descriptive statistics were reported. Patients were divided into the “SJS or SJS/TEN overlap” group when BSA involvement was less than 30%, and “TEN” group with 30% or greater BSA involvement. The more severe eye was chosen for comparison in case of asymmetrical involvement. If a patient was assessed multiple times during the acute phase, the most severe assessment was used for analysis. Comparisons were performed with the chi-square test or Fisher's exact test for categorical data and independent *t*-test for continuous data. A *p*-value of < 0.05 were regarded as statistically significant.

## Results

During the 15-years study period, a total of 56 in-patients were diagnosed with SJS or SJS/TEN overlap (39 cases) or TEN (17 case). 24 patients (19 SJS or SJS/TEN overlap and 5 TEN) were excluded due to the lack of available ophthalmic assessment charts. The main reason was that the initial ocular assessment was performed in the primary referring center, and no further ophthalmic consultations were made after transferring to our center as there was no evidence of ocular involvement. After exclusion, a total of 32 patients were included for the analysis (Table 1). 20 were SJS or SJS/TEN overlap and 12 were TEN patients. The mean age was 44.8 ± 25.0 (range 15–59 years old) for the SJS or SJS/TEN overlap group and 44.3 ± 26.7 (range 3–89 years old) for the TEN group (*p* = 0.96). The male: female ratio was 10:10 and 3:9 respectively (*p* = 0.267). All patients were Chinese, and all cases of SJS or TEN were drug-induced. However, the culprit drug could not be identified in some of the cases, as these patients were given a number of new drugs (several antibiotics, for example) at the same time prior to the disease onset. The visual acuity could not be documented in almost all cases as patients were routinely sedated for pain control in our burn unit during the acute phase of illness.

**Table 1:** This table shows the demographics and specific ocular or systemic treatment received during the acute phase of the Stevens-Johnson syndrome (SJS) and toxic epidermal necrolysis (TEN). All these were comparable between the two groups

	Stevens-Johnson Syndrome and SJS/TEN overlap (*n* = 20)	Toxic Epidermal Necrolysis (*n* = 12)	*p* - value
Age	44.8 ± 25.0	44.3 ± 26.7	0.960
Sex (Male: Female ratio)	10: 10	3: 9	0.267
Intravenous Pulse Steroid	20 (100%)	12 (100%)	1.000
Amniotic Membrane Transplant	1 (5%)	1 (8.33%)	1.000
Intravenous immunoglobulin	11 (55%)	6 (50%)	0.784

The acute ocular manifestations of SJS or SJS/TEN overlap and TEN patients were classified as mild, moderate and severe ocular involvement using the classification system described in the methodology. Overall, the ocular involvement during the acute phase 53% (17 out of 32 patients) in our series, with 40% (8 patients) in the SJS or SJS/TEN overlap group and 75% (9 patients) in the TEN group. Table 2 summarized the findings. For SJS or SJS/TEN overlap patients, 1 (5%) had mild ocular involvement, 3 (15%) had moderate involvement and 4 (20%) had severe involvement. In comparison, for TEN patients, 5 (42%) had mild involvement, 0 (0%) had moderate involvement and 4 (33%) had severe involvement. The TEN group had significantly more mild involvement than the SJS or SJS/TEN overlap group (*p* = 0.01), while in all other groups, no statistically significant differences were found (*p* > 0.05).

**Table 2:** This table shows the severity of ocular involvement during the acute phase in the two groups. The grading system was based on that described by Power et al. [16] There was a statistical significant difference between the degree of ocular involvement in the two groups (0.022). Post-hoc analysis showed that the TEN group had significantly more mild involvement cases than the SJS or SJS/TEN overlap group (*p* = 0.01), while in all other groups, no statistically significant differences were found (*p* > 0.05)

Ocular involvement	Stevens-Johnson syndrome and SJS/TEN overlap	Toxic epidermal necrolysis
None	12 (60%)	3 (25%)
Mild^a^	1 (5%)	5 (42%)
Moderate^b^	3 (15%)	0 (0%)
Severe^c^	4 (20%)	4 (33%)

^a^Mild involvement: lid edema, eyelid skin involvement including denudation and desquamation, mild corneal involvement (punctate fluorescein staining), mild conjunctival injection or chemosis only

^b^Moderate involvement: membranous conjunctivitis, corneal epithelial defects with more than 30% healing with medical treatment, corneal ulceration or corneal infiltrates

^c^Severe involvement: symblepharon formation, acquired eyelid malpositions, non-healing corneal epithelial defects, visual loss or conjunctival fornix foreshortening

We have also graded our series using the system described by Sotozono et al, which graded the cases from grade one to three as shown in Table 3 [17]. For our SJS or SJS/TEN overlap patients, the distribution of grade 1 to 3 was 5% (1 case), 20% (4 cases) and 15% (3 cases) respectively, whereas that for TEN was 42% (5 cases), 17% (2 cases) and 17% (2 cases). Again, the TEN group had significantly more mild involvement than the SJS or SJS/TEN overlap group (*p* = 0.018), while in all other groups, no statistically significant differences were found (*p* > 0.05).

**Table 3:** This table shows the severity of ocular involvement during the acute phase in the two groups, using the grading system based on that described by Sotozono et al. [17] The TEN group had significantly more mild involvement than the SJS or SJS/TEN overlap group (*p* = 0.018), while in all other groups, no statistically significant differences were found (*p* > 0.05)

Grading	Stevens-Johnson syndrome and SJS/TEN overlap	Toxic epidermal necrolysis
0 (None)	12 (60%)	3 (25%)
1 (Mild)^a^	1 (5%)	5 (42%)
2 (Moderate)^b^	4 (20%)	2 (17%)
3 (Severe)^c^	3 (15%)	2 (17%)

^a^Grade 1 (mild involvement): eyes with conjunctival hyperaemia

^b^Grade 2 (moderate involvement): eyes with pseudomembrane formation or presence of epithelial defects

^c^Grade 3 (severe involvement): eyes with both pseudomembrane formation and epithelial defects

For treatment, majority of patients received conventional local therapy, which consisted of frequent topical artificial tears, topical steroids and topical antibiotics. Glass rodding was performed for any signs of early ocular surface adhesions. Systemically, all cases were routinely given early intravenous steroids (1 mg/kg/day for 3 days). In addition, 1 SJS (5%) patient and 1 TEN (8.3%) patient underwent amniotic membrane transplantation (AMT) 5 days after admission (*p* = 1.0). For systemic therapy, other than supportive treatment, 11 SJS or SJS/TEN overlap (55%) and 6 TEN (50%) patients received intravenous immunoglobulin (IVIG) treatment within 5 days after admission (p = 0.784). The overall mortality rate was zero in our series.

## Discussion

Ocular surface disease is a common manifestation of SJS and TEN. We reported an overall 53% acute ocular involvement rate in our series, and this fell into the reported range of 50 to 88% in the literature [6]. Most studies described SJS and TEN as a whole with no comparison between these groups [10, 12, 17–21], with a few exceptions [7, 14, 15]. Morales et al and Gueudry et al classified the cases into 3 categories (SJS, SJS/TEN overlap and TEN) and made comparisons [7, 14], while some studies focused on comparing SJS cases (<10% BSA involvement) with TEN (> = 30% BSA involvement) [15, 17]. In our study, a significant proportion of cases (especially those form the earlier years) only had the diagnosis of SJS (<30%) or TEN (>30%), without clear documentation of the exact percentage of the body surface area involved. Thus, in order to achieve similar categorization to facilitate comparisons between different study results, we grouped all our cases with <30% surface area involvement as a single “SJS or SJS/TEN overlap” group and compared this with the TEN group.

We found a higher proportion of TEN patients with acute ocular surface inflammation than SJS patients (75% in TEN vs 40% in SJS or SJS/TEN overlap), but the difference was not statistically significant. There was also a slightly higher rate of severe ocular involvement in TEN patients (33.3%) compared to SJS or SJS/TEN overlap patients (20%), but the difference was again statistically insignificant. Only in the mild involvement group, the TEN patients had a significantly higher involvement rate than SJS. This was comparable to the study by Yip et al who studied 81 out of 117 Asian patients with acute ocular complications [15]. In their study, TEN patients also had a slightly higher rate of acute ocular involvement compared with SJS, but the difference was statistically insignificant despite adjusting for age and gender. They suggested that the similar mechanisms of apoptosis in the skin and eye in TEN in contrast to SJS patients could be a possible reason for this. In contrary, in the study by Morales et al, they reported a higher prevalence of ocular involvement in the acute phase when the epidermal detachment involved more than 10% of the total body surface area (SJS/TEN overlap and TEN) [7].

Regarding the disease severity classification system, both studies described above employed the same classification described by Power et al. [16] Morales et al reported mild, moderate and severe acute ocular manifestations as 44, 20 and 20% of cases respectively, with no significant difference between the three groups [7]. Similarly, Yip et al reported mild, moderate and severe acute ocular manifestations as 41, 25 and 4% [15]. We initially also employed this classification system as it was commonly used. However, as addressed by Morales et al [7], using this classification in the acute phase had limitations, as the severe category consisted of cicatricial changes (eyelid malpositions and forniceal foreshortening) which only present as chronic manifestations rather than acute ones.

A more recent study by Sotozono et al proposed another grading system for the acute ocular manifestations [17]. This acute ocular severity scoring system mainly focused on the ocular surface inflammation and epithelial necrosis or apoptosis, which they proposed to be the initial ocular pathologic processes of SJS/TEN. The cicatricial changes were also omitted in the grading system. Thus, we have also included this grading system in our results. In their study, the distribution of grade 1 to 3 in SJS was 36, 25 and 17% respectively, whereas that in TEN was 23, 35 and 17% respectively. For our series, the respective distribution was 5, 20 and 15% in SJS group, whereas that for TEN was 42, 17 and 17%. Compared to their results, our SJS or SJS/TEN overlap had less mild involvement, with similar rates in the moderate and severe grade. For TEN, in contrary, we reported more mild involvement cases, but fewer patients with moderate grade. However, as we are grading these cases retrospectively, this might not have reflected the actual distribution.

The treatment for both SJS and TEN had been evolving in the past 16 years. There has been increasing use of early amniotic membrane transplantation (AMT) to treat severe ocular manifestations as several large case series have shown improved ocular outcomes as well as ocular surface histology (Table 4) [19–30]. In the most recent prospective case series published by Gregory, he proposed another acute phase grading system to guide when AMT is indicated [29]. In his study, severe cases which had either corneal epithelial defect beyond punctate keratopathy, or at least 1 lid margin with staining involving more than one-third of its length, or any section of bulbar or palpebral conjunctiva with staining of more than 1 cm in largest diameter, should receive urgent AMT to preserve vision and decrease the risk of ocular surface scarring and dry eyes. For the use of intravenous immunoglobulin (IVIG), the evidence to support its use was contradictory [3]. IVIG did not appear to reduce the severity of visually significant ocular complications in the study by Yip et al [31], whereas another study by Aihara et al, which combined IVIG with corticosteroids, showed beneficial effect in reduction of the severity-of-illness score, as well as improvement in ophthalmic lesions [18]. Using systemic pulse steroid alone at disease onset has also been shown to be beneficial in preventing ocular complications [32]. In our study, all of our cases received early intravenous pulse steroid (1 mg/kg/day for 3 days). We believe this has significantly reduced the severity of the ocular inflammation in our series, and could explain our lower rate of amniotic membrane transplantation. However, the beneficial effect of this universal steroid usage was also a significant confounding factor in this study.

**Table 4:** Case Reports/Studies showing beneficial effects of early AMT during the acute phase of TEN

Study	Year	Study Design	Cases	Severity of Ocular Disease	Age Range (Years old)	Sample Size (Patient)	Significant Findings
John et al [22]	2002	Interventional Case Report	TEN	Severe	6 – 8	2	First use of amniotic membrane transplantation in acute TEN
							First use of amniotic membrane on external eyelid and lid margin
							First to demonstrate of effectiveness of AMT in preventing lid/cornea adhesion and restoring ocular surface integrity
Kobayashi et al [23]	2006	Interventional Case Report	TEN	Severe	6	1	First to show evidence that repeat AMT is effective in treating persistent epithelial defects after initial early AMT
Atzori et al [24]	2006	Interventional Case Report	TEN	Severe	68	1	First report to identify an angiotensin II receptor antagonist as a possible causative agent
Tandon et al [25]	2007	Interventional Case Report	TEN	Severe	12	1	Uses repeated epilation to prevent corneal epitheliopathy in acute and chronic stages
Shammas et al [21]	2009	Retrospective Case Series	SJS or TEN	Severe	2 – 82	8	First to compare Prokera use (partial coverage of ocular surface) and AMT use (complete coverage)
Gregory [19]	2011	Prospective Case Series	SJS or TEN	Severe	3 – 28	10	All patients treated within 10 days of disease onset
							Repeat AMT performed every 10-14 days if ocular surface inflammation persisted
							Further evidence to show that Prokera alone was inadequate
Hsu et al [20]	2012	Case Control Study	SJS or TEN	Mild-Severe	N/A	30 (13 treated with AMT, 17 medical management alone)	Showed better visual outcomes in AMT treated eyes over eyes without AMT across the spectrum of disease
							Showed superior outcomes in eyes with AMT done within the first week of disease onset
Kim et al [26]	2014	Retrospective Case Series	SJS or TEN	Mild-Severe	1 – 59	51	Demonstrated that patients < 18 years of age had worse ocular outcomes
							Demonstrated benefits of IVIG + steroid use on ocular outcomes
Lopez-Garcia et al [27]	2014	Prospective Case Series	TEN	Moderate - Severe	N/A	5	First to show histological evidence to support beneficial effects of AMT
Sharma et al [28]	2016	Randomized Controlled Trial	SJS	N/A (prospective)	31.7 ± 16.7 27.9 ± 12.5	25 + 25	Only prospective randomized control trial evaluating the efficacy of AMT in the management of acute SJS.
Gregory [29]	2016	Prospective Case Series	SJS & TEN	All severity	N/A	79	Proposed a grading system to facilitate decision making on when to do AMT

The main limitation of this study was its retrospective nature. Detailed ocular assessment as suggested by Sotozono was not available during the earlier years of the study, and we were only able to group the manifestations in broad categories [17]. As almost all cases were assessed at the bedside with portable slip lamp and on sedated patients, we were unable to report the visual acuities and the accuracy of the assessment would be limited by the suboptimal setting. Our sample size was also relatively small, as we only included cases with a definite diagnosis of SJS or TEN based on dermatologist’s assessment and skin biopsy. A high exclusion rate (24 out of 56 cases, 42.9%) also contributed to our small sample size. We had a high exclusion rate as these cases had their initial ophthalmic assessment performed in the initial referring center and no further ophthalmic assessment record was available after being transferred to our institution. It was important for us to exclude these cases to avoid misclassifying them as ‘acute stage disease’, since some of these cases might already have many days to weeks of ocular manifestations prior to admission to our burn ICU. Moreover, our center did not adopt using the SCORTEN score until the recent few years. As a result, majority of our retrospective cases did not have the SCORTEN value and we could not perform correlation analysis between the SCORTEN value and the severity of acute ocular involvement. However, it has been recently shown that the SCORTEN value did not correlate with the severity of eye involvement in the acute setting [7]. The lack of a unified categorization method is another major limitation when studying this disease entity. As explained above, we could only group all cases with <30% body surface involvement into a single “SJS or SJS/TEN overlap” group instead of two. Lastly, studies have shown that the severity of involvement in the acute phase did not predict the late ocular outcome [15], and this current study only focused on the acute phase of the disease. However, given the low incidence of the disease, we believe our study would be an important addition to the current literature, especially on Chinese patients.

## Conclusion

Ocular involvement is common during the acute phase in both SJS (40%) and TEN patients (75%) in our series of Chinese patients. TEN patients were more likely to have acute ocular surface inflammation than SJS patients, but the difference was mainly in the mild involvement group. The routine use of early intravenous pulse steroid in all our cases could have explained our low incidence of moderate and severe ocular involvement. Whether the acute ocular manifestation predicts late ocular complications, or whether there would be difference in the chronic ocular manifestations between the SJS or TEN was not explored in this current study. These issues could be further explored in a multicenter prospective study comparing acute ocular manifestations, response to treatment and visual outcomes in SJS and TEN patients, that is currently underway by our investigative team.

## Abbreviations

AMT: Amniotic membrane transplantation
SJS: Stevens-Johnson syndrome
TEN: Toxic epidermal necrolysis
